# Bio-inspired nanomaterials for biomedical innovation

**DOI:** 10.1080/14686996.2020.1786948

**Published:** 2020-07-01

**Authors:** Takanori Akagi, Horacio Cabral, Peng Mi

**Affiliations:** a Department of Material Engineering, School of Engineering, the University of Tokyo, Tokyo, Japan; b Department of Bioengineering, Graduate School of Engineering, the University of Tokyo, Tokyo, Japan; c State Key Lab Biotherapy, West China Hosp, Sichuan University, Chengdu, P. R. China

Nature is a source of inspiration for a wide range of areas in science and technology. In the field of materials sciences, learning from biological principles enables researchers to design and develop advanced materials, structures and tools that are capable of meeting demanding requirements. Bio-inspired nanomaterials are emerging as a promising field of research with a high potential for developing unprecedented approaches for managing human diseases. The significance of these materials is vast, ranging from applications in drug delivery and tissue engineering to sensors and diagnosis devices. Thus, innovation through bio-inspired nanomaterials has allowed great progress in biomedical applications with bio-mimetic capabilities, such as robotic nanodevices, organs-on-chips and tissue engineering. Moreover, recent advances in biotechnology have made it possible to directly isolate and engineer nanomaterials from biological sources, including cellular membranes, organelles and exosomes, as well as to combine them with synthetic nano-materials, providing novel features for advancing the biomedical field.

The potential biomedical functions of the bio-inspired nanomaterials stem from the fact that the human biological system is made up of nanoscale self-assembly biomolecules. As such, the potential therapeutic applications of nanomaterials rely strongly on the similarity of their sizes with the cellular proteins as well as on their additional properties in terms of shape, chemical composition and surface charge. These features determine their intracellular access by endocytosis and by mechanical manners, such as membrane disruption[1].

In this focus issue, we present the progress in the development of bio-inspired stimuli-responsive nanomaterials for delivering bioactive agents. Dr. Gang Liu and his group have critically reviewed the recent progresses in biomimetic nanoparticles for the development of applications in drug targeting and for the emerging applications in theranostics, *i.e*. the synergistic combination of therapy and diagnosis through single platforms[2]. Moreover, Dr. Kanjiro Miyata and his team have presented the development of novel carriers that work as an artificial virus for the delivery of messenger RNA by exploiting the chemical degradability of polymers[3]. Beyond synthetic nanocarriers, Dr. Nobuyoshi Kosaka and Dr. Takahiro Ochiya, together with Dr. Tomofumi Yamamoto, critically reviewed current status of cell-derived exosomes, which are endogenous nanocarriers that can deliver biological information between cells and have distinct biological and physicochemical characteristics that make them unique for developing targeted drug delivery systems[4]. Using biological materials as templates for concrete biomedical applications is also presented in this issue with an accent on the development of agents for pin-point therapies toward improving the biocompatibility and specificity in targeting the diseased tissues. For such purposes, the source of biological materials has its own importance in increasing systemic tolerance and reducing potential inflammation. Thus, Dr. Ebara and his group proposed using viral templates for developing next-generation tumour-targeted agents for applications in the boron neutron capture therapy[5].

We would like to acknowledge all the authors for their outstanding contributions. We certainly believe that these works will provide inspiration to many. Also, we would like to express our sincere gratitude to the staff of *Science and Technology of Advanced Materials* for their kind assistance.
